# Mutation analysis of congenital cataract in a Basotho family identified a new missense allele in *CRYBB2*

**Published:** 2009-07-30

**Authors:** Maneo Emily Mothobi, Shuren Guo, Yuanyuan Liu, Qiang Chen, Ali Said Yussuf, Xinli Zhu, Zheng Fang

**Affiliations:** 1 Center for Gene Diagnosis, Zhongnan Hospital, Wuhan University, China; 2 Health Research and Laboratory Services, Maseru, Lesotho; 3 Department of Anesthesia, Renmin Hospital, Wuhan University, China

## Abstract

**Purpose:**

To identify the causative genetic mutation among the known cataract candidate genes underlying the observed phenotype in a Basotho family, with congenital nuclear cataracts.

**Methods:**

Because of the small family size, we used the functional candidate gene analysis approach. We screened a Basotho family, clinically documented to have congenital nuclear cataracts, for mutation in the candidate genes *CRYG* (*C* & *D*; Crystallin, gamma C and Crystallin, gamma D), *GJA8* (Gap junction protein, alpha 8), *CRY* (*AA* & *AB*; Crystallin, alpha A and Crystallin, alpha B), *CRYBA* (Crystallin, beta A) and *CRY* (*BB1* & *BB2*; Crystallin, beta B1 and Crystallin, beta B2) through polymerase chain reaction analyses and sequencing.

**Results:**

Mutation screening identified only one significant alteration in exon 6 (607G>A) of *CRYBB2*, with a substitution of Valine to Methionine at position 187. This mutation segregated in all five affected family members but it was not observed in any of the unaffected persons of the family. The putative mutation led also to the appearance of a new NIaIII restriction site in the samples of the affected family members that was not present in 100 randomly selected DNA samples from ophthalmologically normal individuals and in 40 unrelated senile cataract patients of the same ethnic background as the family members.

**Conclusions:**

This study identified a missense mutation in *CRYBB2* in a family of Basotho with autosomal dominant congenital cataract (ADCC). In summary, we believe this new missense allele is the probable causative molecular lesion for the observed phenotype in this family.

## Introduction

Cataract is an opacification of the eye lens that frequently results in visual impairment or blindness during infancy and early childhood. On a global scale, cataracts are the leading cause of blindness, accounting for approximately 48% of all blindness [1]. Around three-quarters of the world^’^s blind children live in developing countries in Africa and Asia [2]. Although congenital cataract is much less common than age-related cataract, it is still responsible for approximately one tenth of childhood blindness worldwide [3]. Isolated congenital cataracts tend to be highly penetrant Mendelian traits, with autosomal dominant more common than autosomal recessive and X linked inheritance cataracts [4].

Most progress has been made in identifying the genes causing autosomal dominant congenital cataract [5]. Of the cataract families for whom the mutant gene is known, about half have mutations in crystallins, about a quarter have mutations in connexins, with the remainder divided among the genes for heat shock transcription factor-4 (*HSF4*), aquaporin-0 (*AQP0, MIP*), and beaded filament structural protein-2 (*BFSP2*) [6,7].

Crystallins are known to constitute about 90% of the water-soluble proteins of the lens and contribute to transparency and refractive properties due to a uniform concentration gradient in the lens. The vertebrate crystallins are divided into two families: *α*-crystallins and βγ-crystallins. In addition to being refractive proteins in the lens, *α*-crystallins are molecular chaperones and function as protective proteins against physiological stress [8]. Vertebrate lens βγ-crystallins have two domains, each comprising two structural motifs with signature folds called 'Greek keys' [9]. The relative position of the two domains in γB- and βB2-crystallins (γB and βB2) differs in crystallographic structures, having either ‘closed’ or ‘opened’ conformation, respectively [10]. βγ-Crystallins are more heterogeneous with seven subunits (four acidic βA1-, βA2-, βA3-, and βA4-crystallin, and three basic βB1-, βB2-, and βB3-crystallin) in the heterooligomeric β-crystallin and six subunits (γA-γF-crystallin) in the monomeric γ-crystallin [11].

In the lens, crystallins interact to generate a gradient of refractive index so that light can focus to the retina with minimal scattering. Also protein interactions enhance protein stability or solubility [11]. *β*B2 is the major β-crystallin in the lens [12] and is the least modified during aging [13]. It is also the most soluble of all the β-crystallins, remaining soluble during aging [14], and is needed to maintain the solubility of hetero-oligomers during isolation. There is a tendency for other β-crystallins to precipitate when separated from βB2. This has led to the proposal of a role for βB2 in maintaining the solubility of other β-crystallins that are heavily modified during aging [15].

Crystallins are excellent candidate genes for inherited cataracts because of their high lens expression. Many congenital cataract crystallin mutations have been reported; a missense mutation in *CRYBB2* (Q155X) has been identified in three unrelated families with ADCC [16-18]. Interestingly, the phenotype in each family is very different despite the identical mutation indicating that other modifier genes are likely to influence the cataract phenotype [19]. Another mutation in βB2-crystallin, W151C has been reported recently in human [20] and V187E in mice [21]. Herein, we report the identification (by functional candidate gene analysis) of a novel missense mutation (G607A) in exon 6 of *CRYBB2* that led to an exchange of Val for Met (V187M) as the probable cause of the disease in this family. The majority of visually impaired people live in developing countries, and since most disorders leading to visual impairment are preventable or curable; their control is a priority in these countries. Better understanding of genetic and environmental influences in pathogenesis of congenital and infantile cataract in developing countries may highlight the importance of ophthalmic examination of parents and family members of otherwise healthy children with cataract, to ensure hereditary cases are not overlooked so that genetic counseling may be provided. To our knowledge, this is the first reported case of nuclear cataracts associated with the *CRYBB2* mutation (V187M) affecting a three-generation Basotho family in Lesotho.

## Methods

### Clinical evaluation

**Figure 1:**
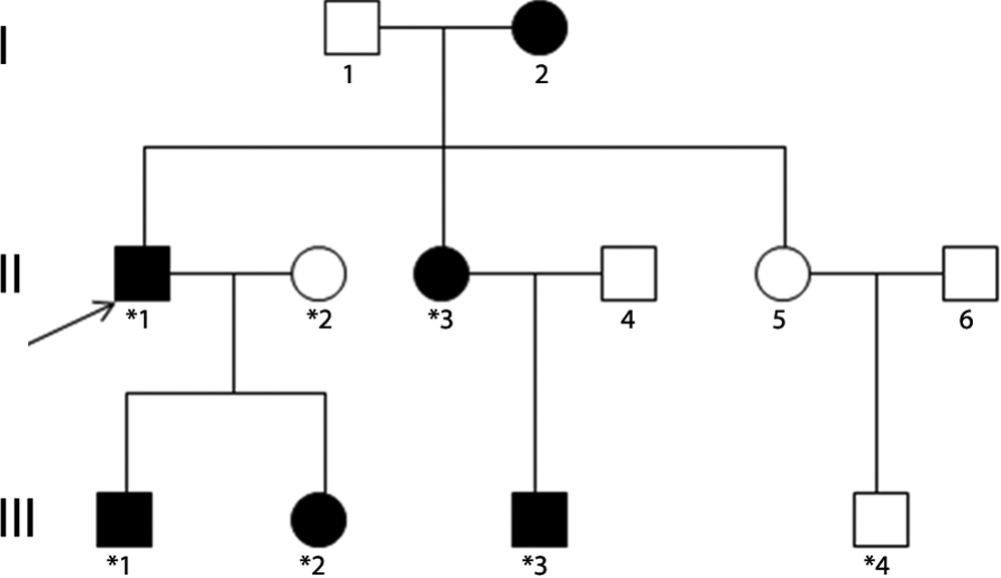
Family pedigree. The family history revealed five affected members in three generations. The dark symbols represent the affected members of the family, while the clear symbols indicate the healthy ones. Circles are for female and squares for male family members. Those who participated in this study are indicated with asterisks. The arrow points to the proband. The pedigree of the family suggests an autosomal dominant mode of inheritance.

The three generation Basotho family (Figure 1) was enrolled from Maluti Adventist Hospital, Lesotho. Written informed consent was obtained from all the participating family members or their parents after explanation of the nature and possible consequence of the study in their native language. A detailed medical history was obtained by interviewing all family members. Cataracts in affected individuals were either present at birth or developed during childhood. Affected status was determined by ophthalmologic examination, which included visual acuity, slit lamp and fundus examination with the dilated pupil. Slit lamp examination of the affected lenses revealed a bilateral nuclear cataract phenotype. No other ocular or systemic abnormalities were found upon physical examination in the affected individuals except for index II:1, III:1 & III:2 (Figure 1), who also presented with strabismus.

Normal subjects and the senile cataract patients matching the ethnic distribution of the family members were recruited from the General Ophthalmology Clinic of the Queen Elizabeth II Hospital, Maseru, Lesotho. The senile cataract patients also underwent full ophthalmologic examination the same way as family members did. Patients with confounding factors such as diabetes or other complicated eye diseases such as glaucoma and uveitis were excluded from this study as the diagnosis criteria. The control subjects were selected randomly during routine medical fitness examination which included visual acuity test. The research was approved by the Zhongnan Hospital Research Ethics Committee and followed the tenets of the Declaration of Helsinki.

### Mutation screening

Blood samples were collected from all the study subjects and spotted (70 µl) on 903^®^ specimen collection paper (control no. A01798; Schleicher & Schuell Inc., Keene, NH). Genomic DNA was isolated using standard proteinase K/phenol method [22]. Briefly, four discs of about 6 mm in diameter were punched out from S&S 903 specimen collection paper stained with dried blood. To prevent DNA contamination, the paper punch was sterilized first by punching out a clean paper at least fifteen times and then by alcohol burner flame after each separate paper punch. The isolated DNA was then amplified by polymerase chain reaction (PCR) for the exons (and their flanking regions) of *CRYGC*, *CRYGD*, *CRYAA*, *CRYAB*, *CRYBA1, CRYBB1, CRYBB2* and *GJA8*. Primers for PCR amplification of exon 6 of the *CRYBB2* were as follows: forward; 5'-CAC TGT GTC CAA GGT CAC ACA GCT AAG C-3' and reverse; 5'-CCC CTC GTT CAC CCT CCC ATC A-3'. The cycling conditions for PCR included a 95 ^o^C preactivation of the enzyme for 3 min, 10 cycles of touchdown PCR with a 1 ^o^C decrement of the annealing temperature per cycle from 70 ^o^C to 61 ^o^C, then maintained at 60 ^o^C for 20 more cycles. Each cycle consisted of a denaturation at 94 ^o^C, annealing step and extension at 72 ^o^C, all for 30 s, with a final extension at 72 ^o^C for 10 min. PCR products obtained from the proband and one unaffected member of the family with these primers were sequenced using ABI Genetic Analyzer 3730 (Invitrogene Ltd, Shanghai, China). The sequencing results were analyzed using Chromas 2.3 and compared with the reference sequence in the NCBI database. The mutation was confirmed by the presence of the cleavage site for the restriction enzyme NIaIII (New England BioLabs, Beijing, China) in the other participating affected family members. For control, we analyzed 100 DNA samples from ophthalmologically normal individuals of the same ethnic background as the family members. The mutation was further analyzed in 40 senile cataract patients by restriction fragment length polymorphism. The resulted fragments were resolved in 8% poly acrylamide gel electrophoresis (PAGE) stained with silver according to the modified protocol of Bassam et al. [23].

### Protein structure analysis

Biophysical predictions of the altered protein were analyzed using the Bioinformatics tool of the Expasy Proteomics server [24]. Secondary structure prediction was carried out by the PHD method [25]. For tertiary structure predictions, we used EsyPred3D [26] and DEEP VIEW/SWISS-Pdb viewer [27] for automated homology protein modeling and visualization of resulting protein database files. For hydropathy analysis we used Kyte-Doolittle hydropathy plots. All hydropathies for both wild type and mutant were calculated in a default window size of 7.

## Results

### Case history

This three generation family included 5 affected individuals with congenital nuclear cataract and 2 unaffected individuals. The proband (II:1), a 44-year-old male, was diagnosed with bilateral cataract at his first decade of life. Later on, his female sib also complained of blurred vision in her early teens. The two children (a girl and a boy) of the proband and the son of the proband’s sister also suffered from congenital cataract from birth. Apart from congenital cataract, the proband and his two children (III:1 & III:2) also presented with strabismus. All the affected individuals in this family underwent cataract extraction so the lens photographs could not be ascertained. A pedigree of the family is given in Figure 1.

### Molecular analysis

**Figure 2:**
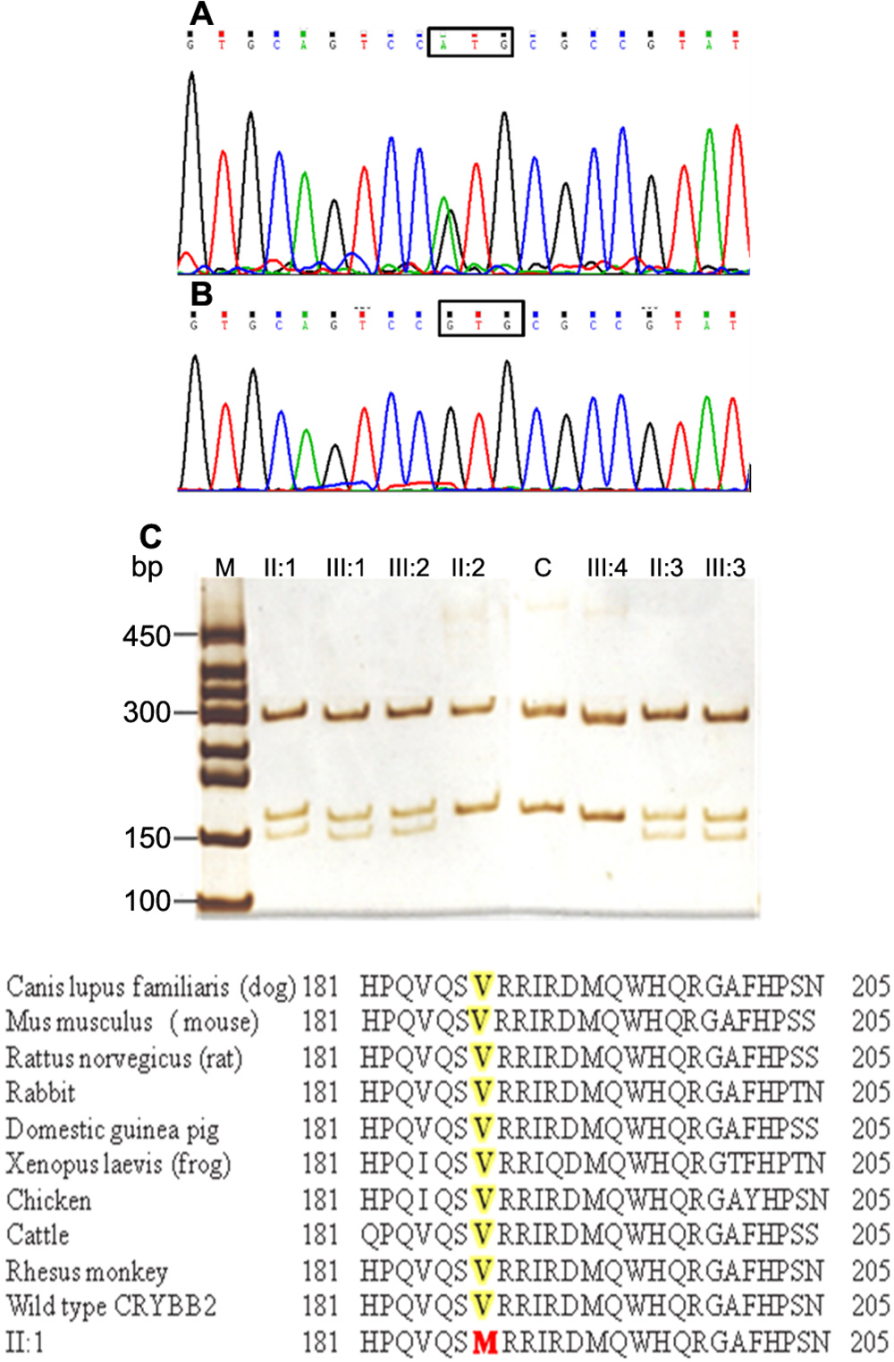
Mutation analysis of *CRYBB2*. The sequence chromatogram of a mutant allele shows a heterozygous G→A transition that changed Valine at codon 187 to Methionine (counting the A of the ATG start codon as number 1; **A**). The sequence chromatogram of a wild type allele shows Valine (GTG) at codon 187(**B**). Restriction fragment length analysis showing that a gain of the novel NIaIII site cosegregated with affected individuals heterozygous with the V187M mutation (300, 168, and 150 bp) but not with unaffected individuals (300 and 168 bp). **C** demonstrates an unrelated healthy individual. **D**: Sequences producing specific alignment of CRYBB2 amino acids. A protein–protein BLAST search (NCBI) of human βB2-crystallin amino acid sequence was done. Proteins having various levels of sequence identity were picked and manually aligned. Shaded letters (yellow) correspond to amino acid that is mutated in this study (highlighted in red).

In the course of functional candidate gene analysis, polymorphic sites, rs2330992 & rs56123451 were identified and since they did not co-segregate with the disease phenotype in this family, they had to be excluded as causative alleles for the mutation. However, bidirectional sequencing of exon 6 of *CRYBB2* in one of the affected individual (II:1) showed a heterozygous change at position 607 (c. 607G→A) (Figure 2A). In contrast, the mutation was not seen in a healthy family member (III:4) (Figure 2B). The mutation was confirmed by an NIaIII digest of the PCR amplified exon 6 of *CRYBB2*. The same alteration was found in the four other family members who were also affected (II:3, III:1, III:2, and III:3) but was not observed in the healthy family members tested nor in the 100 unrelated control subjects and 40 senile cataract patients from the same Basotho population (Figure 2C). This nucleotide substitution replaced an evolutionarily highly conserved Valine with Methionine at amino acid position 187 (p.V187M) in the fourth Greek key motif of βB2 crystallin (Figure 2D).

### Bioinformatics and structure predictions

**Figure 3:**
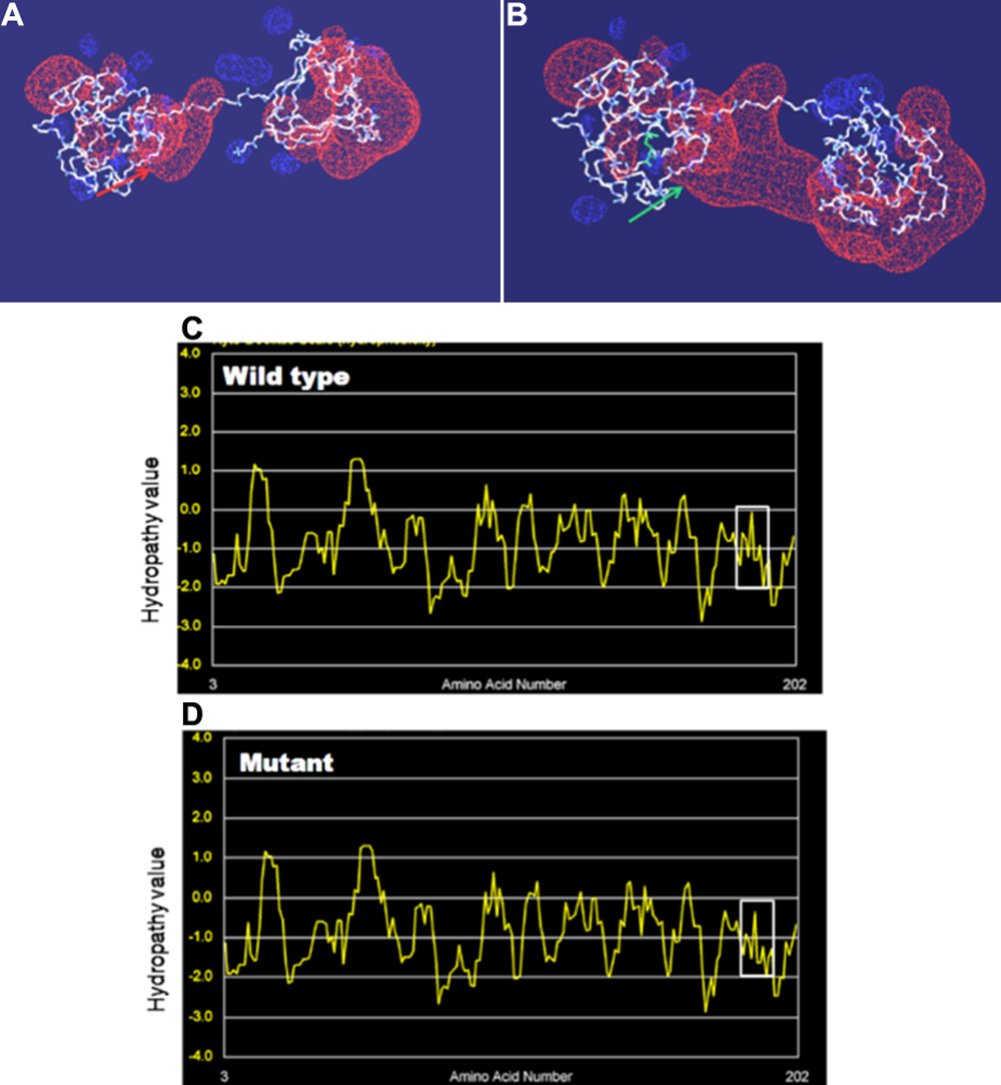
Structural analysis of the mutant protein. The predicted three-dimensional protein structures for CRYBB2^WT^ (**A**) and CRYBB2^V187M^ (**B**), without side chains. The electrostatic potential is displayed in red (negative potential) and blue (positive potential) clouds. The mutant protein exhibits an unusually large negative electrostatic potential (green arrow). The amino acid V187 in the wild type is marked in red and in the mutant form, M187 is marked in green. Kyte-Doolittle hydropathy plot of CRYBB2^WT^ (**C**) and CRYBB2^V187M^ (**D**). X-axis represents position of amino acids. Y-axis represents hydropathy value in a default window size of 7.The region of interest is marked by white boxes. The increase in hydrophilicity in the mutant form is evident.

The secondary structure of the mutant CRYBB2 compared with the wild type was determined by the PHD method which predicted that, the mutant appears to have more α-helical secondary structure content (at amino acid 187 and 192) and less β-strands than the wild-type. In total, 5.85% of the protein is α-helix, all other regions are predicted to be randomly coiled (65.37%) or extended β-strands (28.78%) for the mutant, and 4.88%, 64.88%, and 30.24%, respectively for the wild type. Molecular modeling predicted changes in electrostatic potential that would be expected to reduce the stability of the fourth Greek-key motif. The negative potential around residue M187 in the mutant protein is markedly enlarged (Figure 3A,B). Furthermore, Kyte-Doolittle algorithms for hydrophobicity analysis showed significant variation in the physicochemical properties of the critical region in the mutant form compared with the wild type. The environment surrounding the amino acid “Valine 187” in the wild-type protein is neutral (boxed region). In contrast, in the mutant form the hydropathy environment has become more hydrophilic, with calculated values ranging from 0 to -1.145 (Figure 3C,D).

## Discussion

In the β-crystallins, individual Greek key motifs are encoded by separate exons. The *Cryb* genes consist of six exons: The first exon is not translated, the second exon encodes the NH_2_-terminal extension, and the subsequent four exons are responsible for one Greek key motif each [21]. By molecular screening of β/γ-crystallin genes, in the three generation family of Basotho with ADCC, we finally identified a sequence variation (GA) within exon 6 of *Crybb2*. The corresponding alteration at the amino acid level (Val187Met) is thought to affect the formation of the fourth Greek key motif of the βB2-crystallin. The mutation is located in the (sixteenth β strand) β_4_-sheet of the fourth Greek key motif of the protein, a region crucial to the correct formation of the tertiary structure of the βB2-crystallin, even if previous structural examination of the COOH-terminal region focused on the amino acids from 173 to 185, but did not include Val187 [28]. Improper folding of *CRYBB2,* the most abundantly expressed β-crystallin in the lens, could well cause protein aggregation and result finally in the cataract. Although the function of the Greek key motif is still a matter of debate, computer-based analysis suggests that they form an interdomain association – intramolecular in the -crystallins, and intermolecular in the β-crystallins. In this way, they allow a dense packaging of the proteins minimizing light scattering, but providing an optimum in transparency of the eye lens [29]. βB2-Crystallin is the most abundant β-crystallin in the human lens and is important in formation of the complex interactions of lens crystallins. The *CRYBB2*^V187M^ in this case could have possibly destabilized the complex formation that is critical for lens transparency. A second functional aspect of the Greek key motif is its Ca^2+^ binding properties [29]. In conditions of stress, it may be important for the β-crystalline to be recruited into the cytoplasm to stabilize other proteins via its high beta sheet content, and/or to ensure that storage levels of cytoplasmic Ca^2+^ are maintained [30].

Due to their long life spans, crystallins undergo an unusually large number of modifications with deamidation being a major factor. All modifications except one involve oxidation of either methionine, cysteine, or tryptophan residues. In human congenital cataracts, expression of specific genetic mutations of crystallin genes causes changes in protein–protein interaction and subsequent protein density fluctuations, but at a younger age over a much shorter time period [31]. The *CRYBB2*^V187M^ identified in our family with ADCC results in loss of a valine residue in the fourth motif of the COOH-terminal domain. A protein–protein BLAST search (NCBI) of human βB2-crystallin amino acid sequence shows that valine is absolutely conserved in the COOH-terminal domain (Figure 2D). The removal of a valine residue from a highly conserved structural domain is likely to facilitate further modifications and may contribute to compromised ability of βB2-crystallin to maintain the solubility of other more modified β-crystallins. This is in keeping with the observation by Liu and Liang [32], that mutations in Val-152, Leu-165, and Val-187 destroyed β_13_-, β_14_-, and β_16_-strands located in the motif-4 critical in dimerization and resulting in the large reduction in SEAP activity (reduced protein-protein interation). The β_16_-strand of the fourth Greek key motif is affected in the new allele reported here.

**Figure 4:**
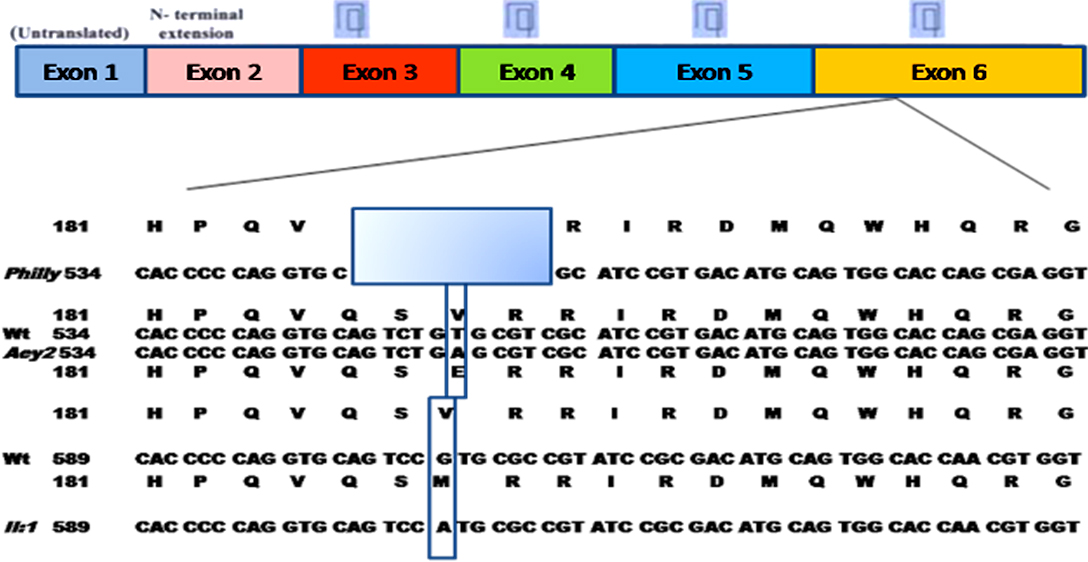
A schematic overview of the mutations identified in *Philly*, *Aey2*, and index II:1 (in our study). The mutation in *Philly* is indicated by a shaded rectangle; the exchanged amino acid is noted below the sequence in *Aey2* and above the sequence in II:1.

Finally, an even more striking consequence is the modification of the electrostatic potential in this area. The mutant protein exhibits a strikingly enlarged negative potential in this region (Figure 3B). This might be the electrostatic explanation for the predicted change of the hydrophobic nature from neutral (0) to -1.145 in this region (Figure 3C,D). It is a matter for speculation whether the increase in hydrophilicity of the altered βB2-crystallin could be due to the oxidation of M187 probably by interacting with water by hydrogen bonding, potentially causing loosening or partial unfolding of the hydrophobic clusters in the protein. Nevertheless, further physico-chemical experiments are necessary to explore the underlying molecular and biochemical mechanisms in details. A corresponding mouse counterpart to the human mutants at the *Crybb2* gene locus is the *Philly* and *Crybb2*^Aey2^ mutations which lead to a progressive dominant cataract although the progression was slower in the *Aey2* mutant than in the *Philly* probably because of the smaller molecular lesion in the *Aey2* mice [21]. The mutation in our family affects also the same region of the protein (close to the carboxyl-terminus) as does the *Crybb2*^Aey2^ mutation (Figure 4).

In conclusion, we identified a missense mutation in *CRYBB2* (V187M) that is associated with autosomal dominant nuclear congenital cataract in a three generation Basotho family. The V187M substitution is thought to affect the formation of the fourth Greek key motif of the βB2-crystallin, potentially disturbing the integrity of the subunit interaction domains which is important in sustaining their stability and or dimerization. However, the mechanisms underlying the molecular process of cataract formation still remain unclear and need further study.
